# Supplementary material for: Risk factors and clinical features for pulmonary paragonimiasis-associated pneumothorax
Source: PLoS Negl Trop Dis. 2023 Dec 15;17(12):e0011828. doi: 10.1371/journal.pntd.0011828 (PMC10756554; doi:10.1371/journal.pntd.0011828)
Supplement: Attachment — Submitted filename: Response to Reviewers_revision1.docx [file pntd.0011828.s002.docx]

September. 12. 2023

Dear, Richard Stewart Bradbury, PhD

Academic Editor

Dear, Uriel Koziol

Section Editor

PLOS Neglected Tropical Diseases

We thank the editors and reviewers of *PLOS Neglected Tropical Diseases* for their review of our article. Our responses to the comments and questions are as follows.

**Manuscript Number: PNTD-D-23-01008**

**Manuscript Title:** **Risk factors and clinical features for pulmonary paragonimiasis-associated pneumothorax**

Comments of the handling editor and the reviewers:

**[REVIEWER COMMENTS]**

**<Methods>**

Reviewer #1:

1. The authors declare to evaluate frequency of paragonimiasis associated pneumothorax and assess risk factors.

2. The study design is inappropriate to test this hypothesis, as it is stated by the authors.

The authors should elaborate more on study methods: The study was stopped in 2017. was there a reason for that or it was arbitrary? further, the authors should expand on the definition of pulmonary paragonimiasis criteria and therefore inclusion criteria in their study.

**▶ Response:** We expected this study to be submitted to a journal in the field of tropical diseases in 2019, but the researchers faced personal family problems, health problems, and school problems. Additionally, the majority of researchers suffered from COVID-19 infection and its aftereffects, which prevented our research from proceeding as smoothly as we had originally intended.

As mentioned in the limitation section, we were unable to confirm all patients with paragonimiasis. Based on relevant literature, inclusion criteria for paragonimiasis includes a positive result in ELISA or confirmation of eggs or flukes in the specimen or tissue. Respiratory symptoms and elevated eosinophile levels, along with exposure to a host related to paragonimiasis, are not specific to the diagnosis. Although, we suspected the possibility of paragonimiasis in some patients, we did not include them in the study unless they clearly met the confirmatory inclusion criteria mentioned above.

We extended the study period to December 2021, adding 47 subjects with paragonimiasis. We considered designing the study as a retrospective cohort study, but paragonimiasis disease is rare and pneumothorax does not commonly occur in paragonimiasis patients. Accordingly, we decided to use a case control study as the study design. (page 6, line 126-127; page 9, line196, 190-200; page 11, 218, 223; page 12, line 232-233; page 13, line 236-243; page 16, line 254-259)

Reviewer #2:

The objectives are clearly articulated and the study design is appropriate. Statistical analysis was correctly used. No ethical or regulatory concerns were noted.

Reviewer #3: (No Response)

**<Results>**

Reviewer #1:

1. How was copd and asthma defined? were on treatment? only self reported? what was the cause, if available, or previous pneumothoraces?

**▶ Response:** Past medical diseases, including COPD and asthma, were confirmed through medical records. We checked the ICD code assigned to each patient that corresponds with each disease for all underlying diseases listed in Table 1.

Previous pneumothorax was confirmed in a total of four patients. The first had pneumothorax when paragonimiasis was diagnosed 12 years ago and was completely cured. A serologic test performed 5 years later showed a negative result for paragonimiasis. In the second case, pneumothorax occurred due to PTNB procedure two years ago. The third case had secondary spontaneous pneumothorax two years ago in association with COPD and lung cancer. The fourth case had primary spontaneous pneumothorax 10 years ago.

2. Univariate analysis should be expanded on table 4 along with collineraity data. a separate table 5 with multivariable analysis of all variables even not significant ones should be created.

**▶ Response:** Table 4 has been modified to include variables removed due to multi-collinearity. Table 5 was also modified in the manner suggested by the Reviewer.

3. Collins and rea methodology for quantity of pleural effusion does not add anything in the manuscript and should be removed.

▶ Response: We agree and have removed the Collins and Rea methodology from the Method section, Results section, and Table 3. (page 7, line147-156; page 13, line 240-241)

Reviewer #2:

Results were clearly presented and supported by appropriate tables. May I suggest to consider renaming "incidence" as "positivity rate". Incidence is more appropriately used when considering a population under follow-up. Please elaborate on the possible reasons for relapse.

**▶ Response:** We have changed ‘incidence’ to ‘positive rate’ as suggested. (page 3, line 63-64; page 19, line 317-319)

**Reviewer #3: (No Response)**

**<Conclusions>**

Reviewer #1:

The discussion section should be enriched. although these data are of significance and population stuydied is significant, the authors should expand on the cause of pneumothorax in their cases. Is it due to asthma, paragonimiasis, or both?

**▶ Response:** We agree with this suggested. According to the Reviewer's opinion, asthma may be the cause of pneumothorax and paragonimiasis may be an independent disease unrelated to pneumothorax. Alternatively, asthma and paragonimiasis may be the cause of concurrent pneumothorax. However, since this study is a case-control study and does not distinguish the content pointed out by the Reviewer, this limitation has been added to the Discussion section. (page 21, line 368-369)

What possible implications have their findings in public health? Future implications should be included in the discussion.

**▶ Response:** We have added information concerning the public health implications (page 20-21, line 335-358).

Reviewer #2:

The conclusions are supported by the data presented. Limitations were properly cited. The manuscript discussed that the data are helpful to advance the understanding of the pneumothorax in pulmonary paragonimiasis.

**Reviewer #3: (No Response)**

**<Editorial and Data Presentation Modifications?>**

Reviewer #1:

a better grammar is also needed. major revision required.

**▶ Response:** Our prior draft had received editing from qualified English-language editors from the Jeonbuk National University Writing Center. The revised draft has also received proofreading, confirmation of which is attached.

Reviewer #2: (No Response)

Reviewer #3: (No Response)

**<Summary and General Comments>**

Reviewer #1:

This is a retrospective study with significant amount of paragonimiasis cases. Although results are interesting and the literature is well covered, i believe significant improvements should be made at methods section, results section and at discussion section., as point by point described above.

**▶ Response:** We have responded to this Reviewer's comment by altering language in the Methods, Results, and Discussion sections.

Reviewer #2:

The manuscript is generally well written. The study assessed the clinical characteristics and risk factors of patients with pneumothorax associated with pulmonary paragonimiasis. The study highlights chest pain and a history of asthma as clinically significant risk factors. Please include pulmonary tuberculosis among the pulmonary diseases that mimic pulmonary paragonimiasis. A recommendation could be a larger cohort of pulmonary paragonimiasis with or without complications of pneumothorax possible with other centers in country or even in the region.

**▶ Response:** We additionally confirmed a history of concurrent pulmonary tuberculosis, which mimics pulmonary paragonimiasis, and added this to Table 1. Histories of past pulmonary tuberculosis are already presented in Table 1.

Reviewer #3:

In this manuscript, the authors have described the risk factors and clinical features for pulmonary paragonimiasis-associated pneumothorax by a retrospective analysis of patients from their center. The data is important as they have touched upon a specific clinical feature of paragonimiasis, although the number of patients is low. However, since it is difficult to have more patients with this complication of pulmonary paragonimiasis, the number of patients is reasonable.

**[EDITOR COMMENTS]**

**The presentation of the data can be improved. The following are my suggestions.**

Line 63-64: Modify as "... the cumulative incidence of pneumothorax was 13.6%..."

**▶ Response:** This sentence has been corrected. (page 3, line 63-64)

Line 69-70: The meaning is not clear. Please rephrase.

**▶ Response:** This sentence has been corrected. (page 3, line 70-71)

Line 126-127: The meaning is not clear. What do the authors mean by "All patients (aged ≥19 years) were diagnosed as having paragonimiasis between May 2011 and October 2017"? Please rephrase.

**▶ Response:** This sentence has been corrected. (page 6, line 126-127)

Line 129-130: Please provide details of the ELISA used? Please mention the source of the kit.

How many patients were found positive by each of the modalities - ELISA, detection of eggs, detection of flukes?

**▶ Response:** The ELISA that we used was: ELISA (Genedia Ab ELISA, Green Cross MS, Yongin, Korea). (page 6, line129)

One-hundred seventy-nine subjects showed positive results in the ELISA test, 15 patients had lung fluke or eggs confirmed in their lung tissue as obtained from a lung biopsy, and five people had Paragonimus eggs, as confirmed through cytology performed on BAL, stool, or other samples. (page 9, line 196-199)

Line 174-175: Please delete the statement about average height of the pneumothorax group greater than that of the non-pneumothorax group as it does not convey any useful information.

**▶ Response:** This sentence has now been deleted. (page 9, line 200-202)

Line 193: Please use "eosinophil" instead of "eosinophile". Also change at other places in the manuscript.

**▶ Response:** Eosinophil has been changed to eosinophile throughout the manuscript. (page11, line 223-224)

Line 221-222: Do the authors want to say that "asthma (odds ratio [OR] 13.11 95% confidence interval [CI], 1.79-96.18) and chest pain (OR 7.41 95% CI, 2.22-24.78) were independent risk factors for pulmonary paragonimiasis-associated pneumothorax" or that asthma in patients with paragonimiasis has higher odds for developing pneumothorax. Similary, a patient of paragonimiasis with chest pain has higher odds of having developed pneumothorax?

Similarly, in the multivariate analysis, the other conditions were also found to have higher odds, i.e., previous pneumothorax and intrapulmonary lesion. These can also be mentioned in the text.

**▶ Response:** We have now described other variables with high odds (previous pneumothorax, intrapulmonary lesions) in the Results section. (page 16, line 254-257)

Line 238-239: Please rephrase.

**▶ Response:** We have made the suggested correction. (page 18, line 285-286)

Line 253: Please replace "larvae" with metacercariae or worm, as appropriate. Also change at other places in the manuscript.

**▶ Response:** This has been corrected. (page 18, line 292, 300, 306)
