# Supplementary material for: Risk factors and clinical features for pulmonary paragonimiasis-associated pneumothorax
Source: PLoS Negl Trop Dis. 2023 Dec 15;17(12):e0011828. doi: 10.1371/journal.pntd.0011828 (PMC10756554; doi:10.1371/journal.pntd.0011828)
Supplement: Attachment — Submitted filename: Response to Reviewers_revision2.docx [file pntd.0011828.s003.docx]

November. 23. 2023

Dear, Richard Stewart Bradbury, PhD

Academic Editor

Dear, Uriel Koziol

Section Editor

PLOS Neglected Tropical Diseases

We thank the editors and reviewers of *PLOS Neglected Tropical Diseases* for their review of our article. Our responses to the comments and questions are as follows.

**Manuscript Number: PNTD-D-23-01008R1**

**Manuscript Title:** **Risk factors and clinical features for pulmonary paragonimiasis-associated pneumothorax**

Comments of the handling editor and the reviewers:

This revised version of the manuscript is much improved. We particularly commend the authors on extending the retrospective study dates to 2021. Some minor elements still require minor amendment, specifically;

1) Plese use "eosinophil" instead of "eosinophile" throughout

**▶ Response**: Eosinophile has been changed to eosinophil throughout the manuscript. (page 10, line 196; page 15, line224; Table 2; Table 4; Table 5)

2) Lines 291, 299, 305. At the stage of the life cycle where Paragonimus are migrating to the lungs, they are correctly called "larvae" (stage between excystation of the metacercariae in the duodenum and maturation to adulthood once established in the lung). Please replace "worm" with "larvae" at these three points (we apologise for prior incorrect instruction regarding this).

**▶ Response**: We changed “worm” to “larvae”. This has been corrected. (page 17, line 262; line 270; line 276)

3) Line 322 please change to "the giant intestinal roundworm"

**▶ Response**: We changed “the round worm” to “the giant intestinal round worm”. This has been corrected. (page 18, line 293)

4) Please italicise genus and species names in your reference list

**▶ Response**: Genus and species names in our reference have been changed to italics. (Reference 1, 3, 21, 22)
